# Composition of Human Skin Microbiota Affects Attractiveness to Malaria Mosquitoes

**DOI:** 10.1371/journal.pone.0028991

**Published:** 2011-12-28

**Authors:** Niels O. Verhulst, Yu Tong Qiu, Hans Beijleveld, Chris Maliepaard, Dan Knights, Stefan Schulz, Donna Berg-Lyons, Christian L. Lauber, Willem Verduijn, Geert W. Haasnoot, Roland Mumm, Harro J. Bouwmeester, Frans H. J. Claas, Marcel Dicke, Joop J. A. van Loon, Willem Takken, Rob Knight, Renate C. Smallegange

**Affiliations:** 1 Laboratory of Entomology, Wageningen University and Research Centre, Wageningen, The Netherlands; 2 Sub-department of Environmental Technology, Wageningen University and Research Centre, Wageningen, The Netherlands; 3 Wageningen UR Plant Breeding, Wageningen University and Research Centre, Wageningen, The Netherlands; 4 Department of Computer Science, University of Colorado, Boulder, Colorado, United States of America; 5 Institut für Organische Chemie, Technische Universität Braunschweig, Braunschweig, Germany; 6 Cooperative Institute for Research in Environmental Sciences, University of Colorado, Boulder, Colorado, United States of America; 7 Department of Immunohematology and Blood Transfusion, Leiden University, Leiden, The Netherlands; 8 Business Unit Bioscience, Plant Research International, Wageningen University and Research Centre, Wageningen, The Netherlands; 9 Centre for BioSystems Genomics, Wageningen, The Netherlands; 10 Laboratory of Plant Physiology, Wageningen University and Research Centre, Wageningen, The Netherlands; 11 Howard Hughes Medical Institute, Boulder, Colorado, United States of America; Global Viral Forecasting Initiative, United States of America

## Abstract

The African malaria mosquito *Anopheles gambiae sensu stricto* continues to play an important role in malaria transmission, which is aggravated by its high degree of anthropophily, making it among the foremost vectors of this disease. In the current study we set out to unravel the strong association between this mosquito species and human beings, as it is determined by odorant cues derived from the human skin. Microbial communities on the skin play key roles in the production of human body odour. We demonstrate that the composition of the skin microbiota affects the degree of attractiveness of human beings to this mosquito species. Bacterial plate counts and 16S rRNA sequencing revealed that individuals that are highly attractive to *An. gambiae s.s.* have a significantly higher abundance, but lower diversity of bacteria on their skin than individuals that are poorly attractive. Bacterial genera that are correlated with the relative degree of attractiveness to mosquitoes were identified. The discovery of the connection between skin microbial populations and attractiveness to mosquitoes may lead to the development of new mosquito attractants and personalized methods for protection against vectors of malaria and other infectious diseases.

## Introduction

Host location by female mosquitoes is mediated by host-derived physical and chemical cues. Physical cues include heat, moisture and visual cues, and play a role during orientation and landing [1], [2], [3]. Chemical cues are considered most important for orientation and landing, especially for nocturnal mosquitoes [4], [5] and humans can be ranked for attractiveness to mosquitoes by testing the emanations released from their skin [6], [7], [8], [9]. The mosquito *Anopheles gambiae* Giles *sensu stricto* (hereafter *An. gambiae*), a nocturnal, highly anthropophilic species, is one of the most important malaria vectors in Africa [5]. Volatiles released from human skin provide essential cues that guide this mosquito species to its host [4], [5].

Skin bacteria play an important role in the production of human body odour and without bacteria, human sweat is odourless to the human nose [10]. Skin bacteria convert non-volatile compounds into volatile compounds having characteristic smells. The body odour of individual human beings correlates with the presence of specific microorganisms [11], [12], [13] and with detailed skin microbial profiles, as assessed using denaturing gradient gel electrophoresis (DGGE) analysis [14]. The interactions between skin microbes and the human host, however, are still poorly understood [15] and the effect of the skin microbial composition on disease vectors remains largely unknown [16].

Human eccrine sweat is more attractive to *An. gambiae* after incubation with skin bacteria for one or two days [17], and washing the feet with a bactericidal soap significantly alters the selection of biting sites by *An. gambiae* [18]. Recently, it was shown that volatiles produced by human skin bacteria, grown *in vitro*, are attractive to female *An. gambiae* when tested in an olfactometer or with mosquito traps [19], [20].

Here, we examined *in vivo* how the composition of skin microbiota affects an individual's attractiveness to mosquitoes by assessing the attractiveness of 48 human males to *An. gambiae* and analysing their skin bacterial communities. We subsequently correlated the observed effects with microbial abundance and composition.

## Methods

### Mosquitoes

The *Anopheles gambiae* Giles *sensu stricto* colony originated from Suakoko, Liberia. Mosquitoes were reared according to the methods described previously [9].

### Volunteers

The attractiveness of 48 adult males aged between 20 and 64 years to *An. gambiae* was examined. Forty-six men were of Caucasian origin, one man was of Asian and one of Hispanic origin. The Dutch Medical Ethical Review committee (METC, Project number ABR NL16928.081.07 amended in 2007) approved the study, and written informed consent was acquired from all subjects prior to participation. Volunteers were requested to refrain from drinking alcohol [21], eating garlic, onions or spicy food, taking a shower, using perfumed cosmetics and were asked to wear nylon socks provided by the research team for the twenty-four hours before the sampling event. Volunteers were free from chronic illnesses and not using any medication on a regular basis. The socks provided by the research team (100% polyamide, 40 denier, Hans Textiel, The Netherlands) were washed twice with 70% ethanol and dried in a ventilated oven at 80°C before use. Volunteers were instructed not to use soap the last time they showered before the experiment.

### Olfactometer bioassay

Skin emanations from each individual were collected twice on three different days by rubbing six glass beads [9] (15 mm in diameter, contained in a Teflon holder, Figure S1) for 10 min. against the underside of the left foot. Feet produce volatiles that are attractive to *An. gambiae* and there is evidence that this body part produces volatiles that influence the selection of biting sites by this mosquito species [18]. Beads with skin emanations were tested for attractiveness to female *An. gambiae* in a dual-choice olfactometer (1.60×0.66×0.43 m) against a standard ammonia concentration of 136 ppm for six times in total: two consecutive assays on each of three mornings [9]. Release of test stimuli was alternated between left and right ports of the trapping devices of the olfactometer to rule out any positional effects. Air speed at the ports was 0.21±0.01 m/s. The experimental room was maintained at a temperature of 27.9±0.7°C and a relative humidity of 62.3±5.8%. The temperature inside the flight chamber was 27.9±1.7°C and the humidity 69.0±4.6%. The humidity of the air led into the trapping devices was maintained above 80% and its temperature was 28.0±1.5°C.After use, the trapping devices were washed in a dishwasher at 45°C with biological soap (Sonnett tabs, Sonnet OHG, Germany). The glass beads were cleaned by rinsing in a solution of 10% Helmanex® II cleaning concentrate (Hellma GmbG & Co KG, Germany) in water, subsequently in distilled water, and finally in ethanol (99.8% purity; Merck, Germany). The rinsed beads were dried in an oven at 200°C for at least one h. Between experiments, the Teflon holder was cleaned with 70% ethanol and quick-dried with a heat gun (Ferm B.V., The Netherlands).

### Skin bacterial diversity

The skin bacterial composition on the feet of the individuals was determined by selective and non-selective plate counts and 16S rRNA gene sequencing. Connecting the selective and non-selective plate counts to the relative attractiveness of the skin volatiles provided a first indication of whether the skin microbiota affects the attractiveness of human skin emanations to *An. gambiae*. The 16S rRNA genes contain hypervariable regions, the sequences of which can provide a detailed signature of the microbiota on the human foot.

#### Sample collection

On each experimental day a bacterial sample was taken from the sole of the left foot of each individual after assessing that individual's attractiveness to mosquitoes during two successive olfactometer experiments. Bacterial samples were taken by using a sampling ring and washing buffer as described before [12], [19]. Seven hundred µL of the sample was added to 300 µL glycerol (87%, Merck, Germany) and stored at −80°C for later identification by 16S rRNA sequencing. The remainder of the sample was used for plate counts on selective and non-selective media.

#### Selective and non-selective plate counts

Within three hours after the bacterial sample collection, 100 µL of each sample was decimally diluted, spread on Colombia (sheep) blood agar plates (Tritium, The Netherlands) and incubated at skin temperature (34°C) to determine bacterial densities by counting colony-forming units (CFU). A range of selective media was used to determine the diversity of the human skin microbiota samples according to the method described before [8] (Table S1). Media were either selective for staphylococci, aerobic corynebacteria, micrococci or Propionibacteria (Tritium, The Netherlands).

#### Bacterial 16S rRNA genes

In total 144 bacterial samples were collected; three samples from each volunteer on different mornings. Microbial DNA was extracted from 41 bacterial samples using a FastPrepDNA soil kit (MP Biomedicals, USA). Of these, 13 samples did not yield enough DNA and were therefore excluded from further analysis. The remaining 116 samples were extracted using the Mo-Bio Power Soil kit (MO BIO Laboratories, Inc., USA). Results of the two extraction methods were not different and the data were combined for the final analysis.

PCR amplification of the V2 region of bacterial 16S rRNA genes, amplicon quantification, pooling, and pyrosequencing were performed as previously described [22]. Sequences were submitted to the MG-RAST database (http://metagenomics.anl.gov/) under the study number qiime:814.

Post-processing of the pyrosequencing output was performed with the QIIME software package [23]. First appropriate denoising of the 454 pyrosequencing output was performed using the PyroNoise algorithm [24]. Then the UCLUST software (http://www.drive5.com/usearch/usearch.pdf) was used to pick clusters of operational taxonomic units (OTUs) at the 97% similarity level. The Ribosomal Database Project (RDP) classifier software [25], with the default training taxonomy, assigned taxonomic labels to the resulting OTUs. Finally, the OTUs were placed in a *de novo* phylogenetic tree with FastTree 2 [26].

### Statistical Analysis

#### Olfactometer data

A GLM (Generalized Linear Model; binomial, logit link function, dispersion estimated, Genstat version 13.2) was used to investigate differences in relative attractiveness between individuals, expressed as the fraction of mosquitoes caught in the trapping device baited with the glass beads releasing odour of the individual being tested divided by the total number of mosquitoes trapped in the two trapping devices together [9]. The GLM was followed by a *t*-test to calculate pairwise differences between means. Individuals were classified as highly attractive (HA) when their mean relative attractiveness was significantly higher than the mean relative attractiveness of each individual in the group classified as poorly attractive (PA) which means the standard error of the GLM parameter estimate did not overlap. Effects were considered to be significant at *p*<0.05 [9].

#### Skin bacterial diversity

The effect of average bacterial densities, expressed as the logarithm of the counts of CFUs on the selective and non-selective plates, on the relative attractiveness of the individuals was analysed using a GLM (Binomial, logit link function, dispersion estimated, Genstat version 13.2).

The 16S rRNA sequence results were used to determine the diversity of skin bacterial communities of the individuals. A phylogenetic diversity (PD) test [27], was used to compare the branch length of the parts of the phylogenetic tree covered by the samples. To control for sequencing effort, multiple rarefaction analyses [28] were performed on all samples at various sequencing depths (1,000 rarefaction samples at each sequencing depth from 500 to 3,000 at intervals of 500). A *t*-test was used (R programming environment, http://www.R-project.org) to test for a significant difference in microbial diversity between the PA and HA group at different sampling depths. No statistics were performed for the diversity at sequence depths above 1500 because the samples of some individuals did not contain more than 1500 sequences.

Based on previous results of *in vitro* experiments, an ANOVA (R programming environment) test was performed to determine whether the observed variance in the relative abundances of five specific genera [20] was partitioned according to the PA and HA group. The data were controlled for sequencing effort. A thousand stochastic rarefactions of the data were used at a simulated sequencing depth of 1500. An ANOVA was performed on each of those rarefied OTU-tables and the median *P*-values determined.

The relative abundances of the genera identified in the skin bacterial samples of the PA and HA individuals were analysed by multivariate partial least squares discriminant analysis (PLS-DA; SIMCA-P 12.0, Umetrics, Sweden) [29], [30]. The data were log-transformed and scaled to unit variance. The number of significant PLS components was determined by cross-validation and the model validated by permutation testing [29], [31]. To avoid over-parameterization of the model, genera with a minor contribution to the discrimination of the two groups in a first PLS-DA were excluded from the final model (variable influence on projection value<1 [26], [28]).

## Results

**Figure 1:**
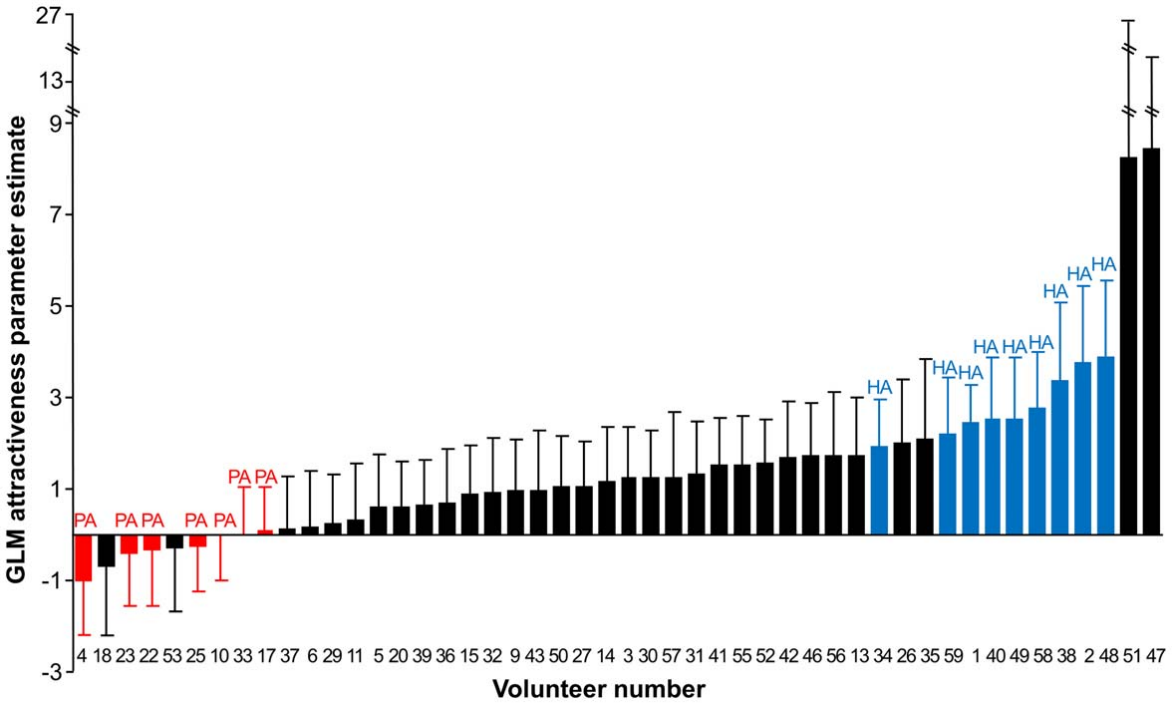
Relative attractiveness to *An. gambiae* of 48 individuals. Bars show the attractiveness parameter estimate results from the Generalized Linear Model (GLM) used to investigate the relative attractiveness [9] of each individual to *An. gambiae*. Individuals were classified as highly attractive (HA, blue bars) when their mean relative attractiveness was significantly higher than the mean relative attractiveness of each individual in the group classified as poorly attractive (PA, red bars) (GLM, *p*<0.05). Error bars represent the standard error of the mean from six replications.

The relative attractiveness of the individuals to *An. gambiae* was significantly different (*P*<0.001; GLM). Nine out of the 48 individuals were significantly more attractive (Highly Attractive, HA group, Figure 1) than seven other individuals (Poorly Attractive, PA group, *p*<0.05; GLM, Figure 1).

**Figure 2:**
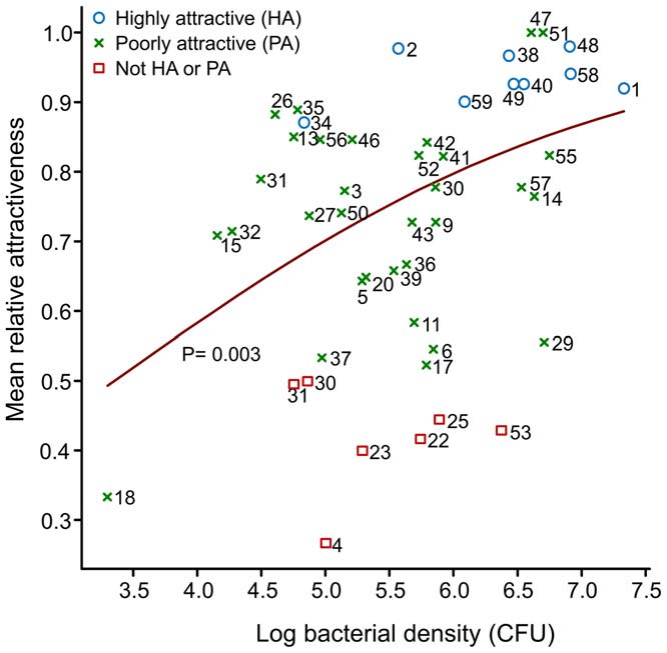
Skin bacterial abundance and relative attractiveness to *An. gambiae*. Correlation between the number of bacteria (log), determined by counts of colony forming units (CFUs) on non-selective plates and the relative attractiveness of the individuals. The relative attractiveness is expressed as the number of mosquitoes caught in the trapping device releasing the odour of the tested individual divided by the total number of mosquitoes trapped in both trapping devices [9]. The red line indicates the fitted relationship according to the Generalized Linear Model (GLM).

Non-selective plate counts showed that, on average, 5.8×10^5^ culturable bacteria were present per cm^2^ on the sole of a human foot, as determined by counting colony-forming units (CFUs) on blood agar plates. The average number of bacteria per cm^2^ on the sole of a foot positively correlated with the relative attractiveness of the individuals to *An. gambiae* (*p* = 0.003; GLM; Figure 2). The abundance of *Staphylococcus* spp. was also positively correlated with the relative attractiveness of the individuals to *An. gambiae* (*p* = 0.01; GLM; Figure S2). The number of bacteria per cm^2^ as determined by CFU counts on blood agar plates and the number of colonies per cm^2^ on plates selective for *Staphylococcus* spp. strongly correlated, suggesting that many of the bacterial colonies found on the blood agar plates were *Staphylococcus* spp. The abundance of *Corynebacteria* spp., *Micrococcus* spp. and *Propionibacteria* spp. did not show a correlation with the relative attractiveness of the individuals (*p* = 0.085, *p* = 0.28 and *p* = 0.41, respectively; GLM).

**Figure 3:**
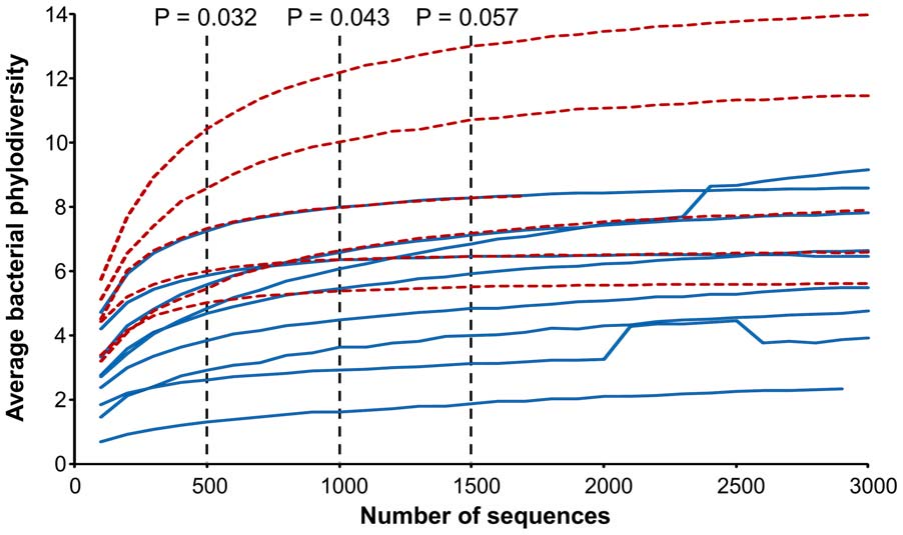
Rarefaction curves showing average bacterial diversity from poorly attractive (PA,) and highly attractive (HA) individuals. *P*-value for the difference in diversity score between PA (dashed red line) and HA (solid blue line) individuals is given at three sampling depths (not calculated for higher numbers of sequences, because the samples from some individuals did not yield more than 1500 sequences).

The 16S rRNA sequence results showed that the phylogenetic diversity (PD) scores [27] of the HA individuals and the PA individuals were significantly different. PD-scores of communities on the PA individuals were significantly higher than those on the HA individuals at a simulated depth of 500 and 1000 sequences and marginally significant at a sequencing depth of 1500 sequences (*p* = 0.032, *P* = 0.043 and *p* = 0.057, respectively; *t*-test; Figure 3). At a sequence depth of 1000, the PD-scores of bacterial communities on the skin of PA individuals were 38% higher than the PD-scores of bacterial communities on the skin of HA individuals.

The relative abundances of OTUs identified by the pyrosequencing procedure and classified within the *Staphylococcus* and *Pseudomonas* genera were significantly different between HA and PA individuals (*p* = 0.024 and *p* = 0.005, respectively; t-test). The abundance of *Staphylococcus* spp. was 2.62 times higher in the HA group than in the PA group and the abundance of *Pseudomonas* spp. 3.11 times higher in the PA group than in the HA group. The abundance of *Brevibacterium* spp. and *Corynebacterium* spp. was not significantly different between the PA and HA group (*p* = 0.52 and *p* = 0.26, respectively; t-test). The effect of the abundance of *Bacillus* spp. on the attractiveness was not tested because they were present in only a limited number of samples.

**Figure 4:**
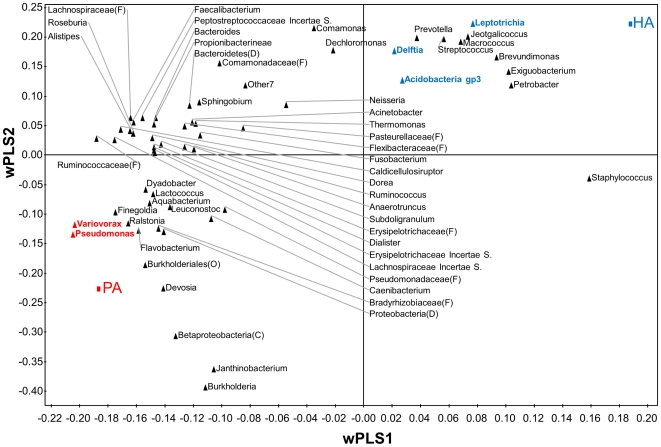
Multivariate data analysis of the bacterial profiles of poorly attractive (PA) and highly attractive (HA) individuals. Partial least squares discriminant analysis (PLS-DA) loading plot based on the relative abundance of bacterial genera in the microbiota profiles of poorly attractive (PA) and highly attractive (HA) individuals. Bacterial genera closer to HA or PA in the plot are more closely correlated to either HA or PA individuals. PLS 1 (*R^2^X* = 0.370, *R^2^Y* = 0.682, *Q^2^* = 0.553) and PLS 2 (*R^2^X* = 0.102, *R^2^Y* = 0.260, *Q^2^* = 0.424) are given. Genera that significantly contribute to the prediction of the model are indicated in blue (HA individuals) and red (PA individuals) (based on 95% confidence intervals, Figure S3). Some sequences could only be identified to division (D), class (C), order (O) or family (F).

Additional genera that correlated with human attractiveness to *An. gambiae* were identified by PLS-DA [29], [30], [31]. The model differentiated the PA and HA groups based on the relative abundances of the bacterial genera (three significant latent variables, *R^2^X_cum_* = 0.544, *R^2^Y_cum_* = 0.993, *Q^2^_cum_* = 0.823). PLS regression coefficients were determined to identify genera that were most characteristic for either group (Figure S3). *Variovorax* spp. and *Pseudomonas* spp. were significantly correlated with PA individuals based on their high PLS regression coefficients (95% confidence interval, Figure 4 and Figure S3). *Leptotrichia* spp., *Delftia* spp. and *Actinobacteria Gp3* spp. were significantly correlated with HA individuals (Figure 4 and Figure S3).

## Discussion

In this study we demonstrated that the composition and abundance of the human skin microbiota influences the relative degree of attractiveness of a human to the malaria mosquito *An. gambiae*. Individuals with a higher microbial diversity were less attractive to the mosquitoes and several bacterial genera were identified that correlated with HA or PA individuals. Identification of the volatiles produced by these genera is likely to lead to the development of new mosquito attractants or repellents [20].

Only a small part of the bacteria found on the human skin are culturable [32] and therefore it was an important confirmative finding that the results from our *in vivo* study corroborated previous *in vitro* studies in which volatiles released by *Staphylococcus epidermidis* were attractive to *An. gambiae* females [19], [20] and volatiles from *Pseudomonas aeruginosa* unattractive [20]. The undirected search by PLS-DA in this study resulted in the identification of several new genera that are correlated with the relative degree of attractiveness of human beings to *An. gambiae*.

The correlation between *Pseudomonas* spp. and PA individuals is in accordance with *in vitro* experiments showing that the blend of compounds produced by *P. aeruginosa* is attractive to *An. gambiae* (in contrast to the volatiles produced by four other bacterial species, all commonly found on human skin) [20]. Our results suggest that *Pseudomonas* spp. and possibly *Variovorax* spp. a) convert some of the attractive compounds produced by other bacteria, b) signal to other bacteria in ways that prevent them from emitting these attractive compounds, c) produce compounds that repel *An. gambiae*, or d) mask the effect of the attractive volatiles emanating from the human skin. More heterogeneous microbiotas may include more bacterial species that produce volatiles attenuating the attractiveness of PA individuals to mosquitoes, and may explain the interference effect described for the yellow fever mosquito *Aedes aegypti* (L). [7]: higher levels of specific volatile compounds were found to be responsible for decreased attractiveness of individuals to *Ae. aegypti*. We hypothesize that lower attractiveness to mosquitoes is caused by a selective group of skin microbiota that emanates compounds that interfere with the attraction of mosquitoes to their human hosts and thus function as an in-built defence system [33]. Genes of the Major Histocompatibility Complex (MHC) have been shown to influence body odour [34], [35], [36] and may exert this influence by changing the skin microbiota composition and hence the volatiles produced by these bacteria and/or the human host [16], [37].

The current study shows that the skin microbiota could play an important role in this built-in defence system and may, therefore, affect transmission of malaria parasites [16], [38]. Individuals with a higher microbial diversity and a higher abundance of *Pseudomonas* spp. or *Variovorax* spp. are less attractive to mosquitoes and may therefore receive fewer bites. Future studies should confirm if individuals with a specific microbiota composition run a lower risk of becoming infected with parasites, and consequently have a higher survival probability.

Compounds that inhibit microbial production of human odour [13], or manipulation of the composition of the skin microbiota may reduce a person's attractiveness to mosquitoes. Analysis of the bacterial volatiles attractive to mosquitoes produced by the *Leptotrichia* spp., *Delftia* spp. and *Actinobacteria Gp3* spp. bacteria identified in this study [20] will also contribute to the development of attractants to be used in traps for monitoring malaria mosquito populations or lure-and-kill strategies [39].

The results presented in this study contribute to our fundamental understanding of the behavioural ecology of mosquitoes. The specialization of *An. gambiae s.s.* on human odours mediated by the composition of the human bacterial community may account for the high degree of anthropophily of *An. gambiae s.s*. Interestingly, the two closely-related sibling species *An. quadriannulatus* and *An. arabiensis*, have a wider host range that is more zoophilic [40], [41], [42] or opportunistic [43], [44], respectively. Bacterial communities of other vertebrate species are likely to differ from those on human beings and may play an important role in determining the host range of mosquitoes [45].

## Supporting Information

Figure S1 **Skin emanation collection.** Teflon holder with six glass beads for collecting skin emanations from human feet to be used for mosquito attractiveness tests in the olfactometer. Distances are given in mm.
(TIF)

Figure S2 ***Staphylococcus*** **spp.-selective plate counts and relative attractiveness to** ***An. gambiae*** **.** Correlation between the number of *Staphylococcus* spp. bacteria (log), determined by counts of colony forming units (CFUs) on *Staphylococcus* spp. selective plates and the mean relative attractiveness of the individuals. The relative attractiveness is expressed as the number of mosquitoes caught in the trapping device releasing the odour of the tested individual divided by the total number of mosquitoes trapped in both trapping devices. The red line indicates the fitted relationship according to the Generalized Linear Model (GLM).
(TIF)

Figure S3 **Coefficient plot of the bacterial profiles of poorly attractive (PA) and highly attractive (HA) individuals.** Partial least squares-discriminant analysis (PLS-DA) coefficient plot based on the relative abundance of bacterial genera in the microbiota profiles of PA and HA individuals. Genera with significantly positive (>0) or negative (<0) PLS regression coefficients (i.e. no overlap between the 95% confidence interval indicated and the horizontal axis) contribute significantly to the prediction of the HA individuals (blue bars) or PA individuals (red bars), respectively. Coefficients were scaled and centred. Some sequences could only be identified to division (D), class (C), order (O) or family (F).
(TIF)

Table S1 **Ingredients of selective media used to determine the diversity of the human skin microbiota samples.**
(DOC)
