# Supplementary figures and images for: Composition of Human Skin Microbiota Affects Attractiveness to Malaria Mosquitoes
Source: PLoS One. 2011 Dec 28;6(12):e28991. doi: 10.1371/journal.pone.0028991 (PMC3247224; doi:10.1371/journal.pone.0028991)

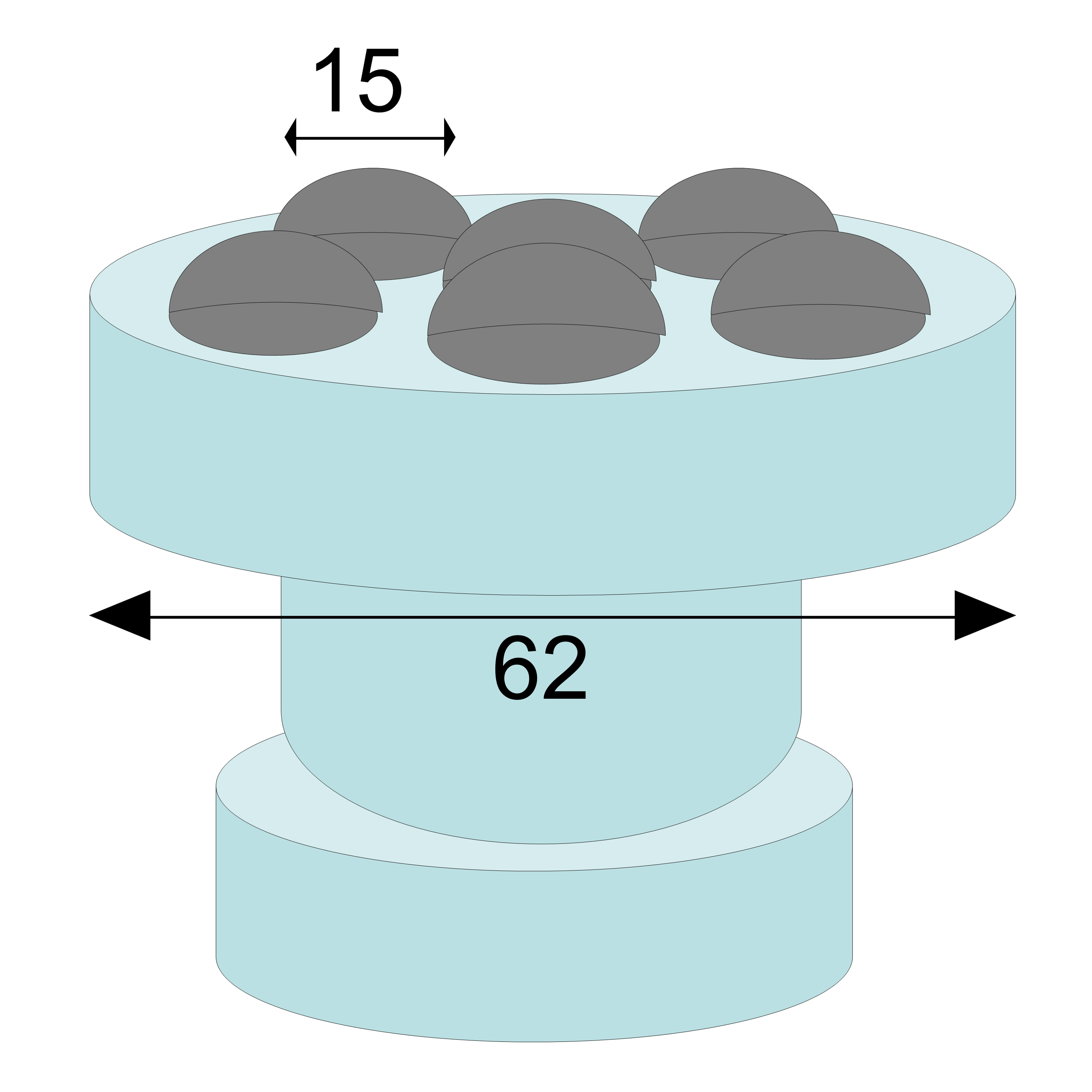

Supplement: Figure S1 — Skin emanation collection. Teflon holder with six glass beads for collecting skin emanations from human feet to be used for mosquito attractiveness tests in the olfactometer. Distances are given in mm. (TIF) [file pone.0028991.s001.tif]

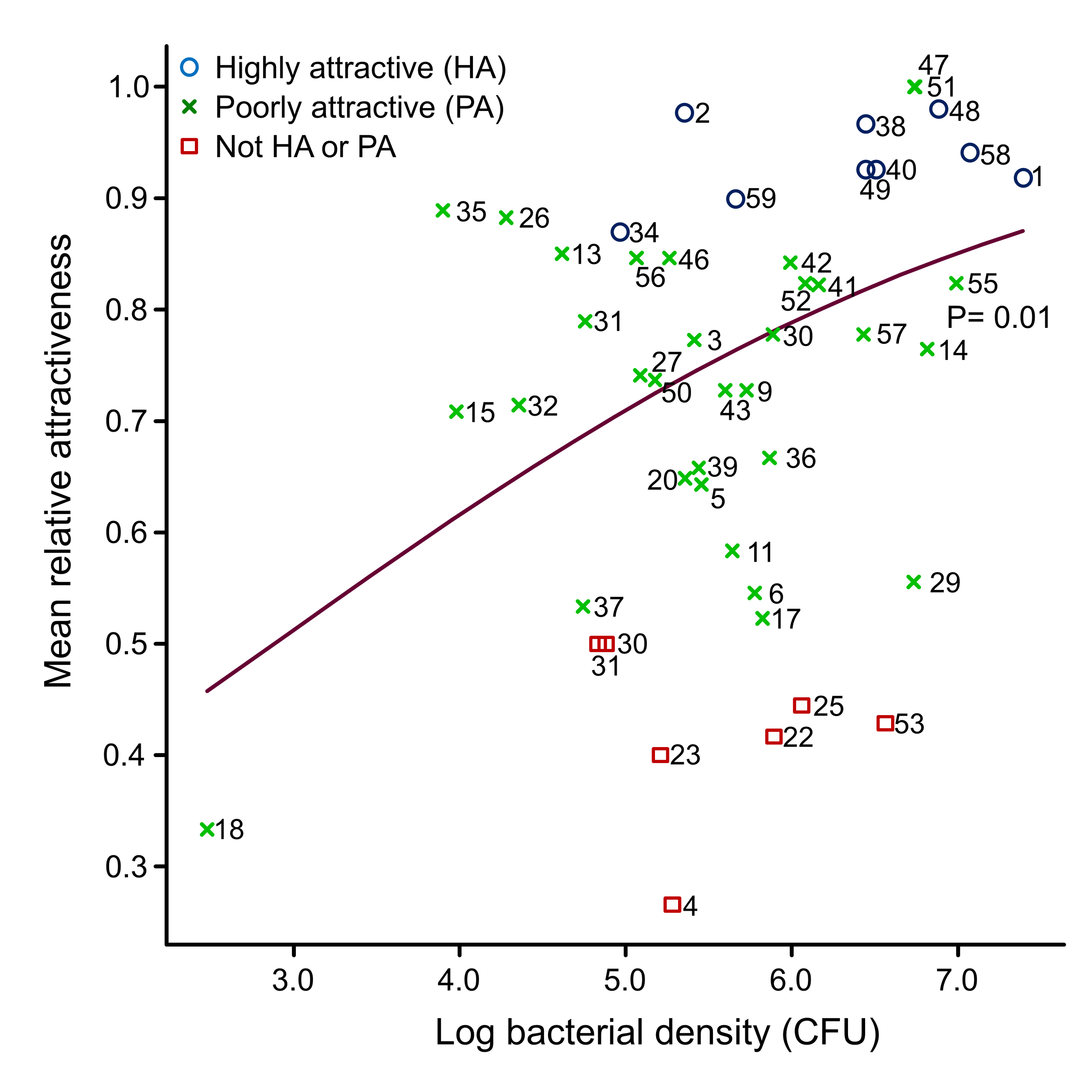

Supplement: Figure S2 — Staphylococcus spp.-selective plate counts and relative attractiveness to An. gambiae . Correlation between the number of Staphylococcus spp. bacteria (log), determined by counts of colony forming units (CFUs) on Staphylococcus spp. selective plates and the mean relative attractiveness of the individuals. The relative attractiveness is expressed as the number of mosquitoes caught in the trapping device releasing the odour of the tested individual divided by the total number of mosquitoes trapped in both trapping devices. The red line indicates the fitted relationship according to the Generalized Linear Model (GLM). (TIF) [file pone.0028991.s002.tif]

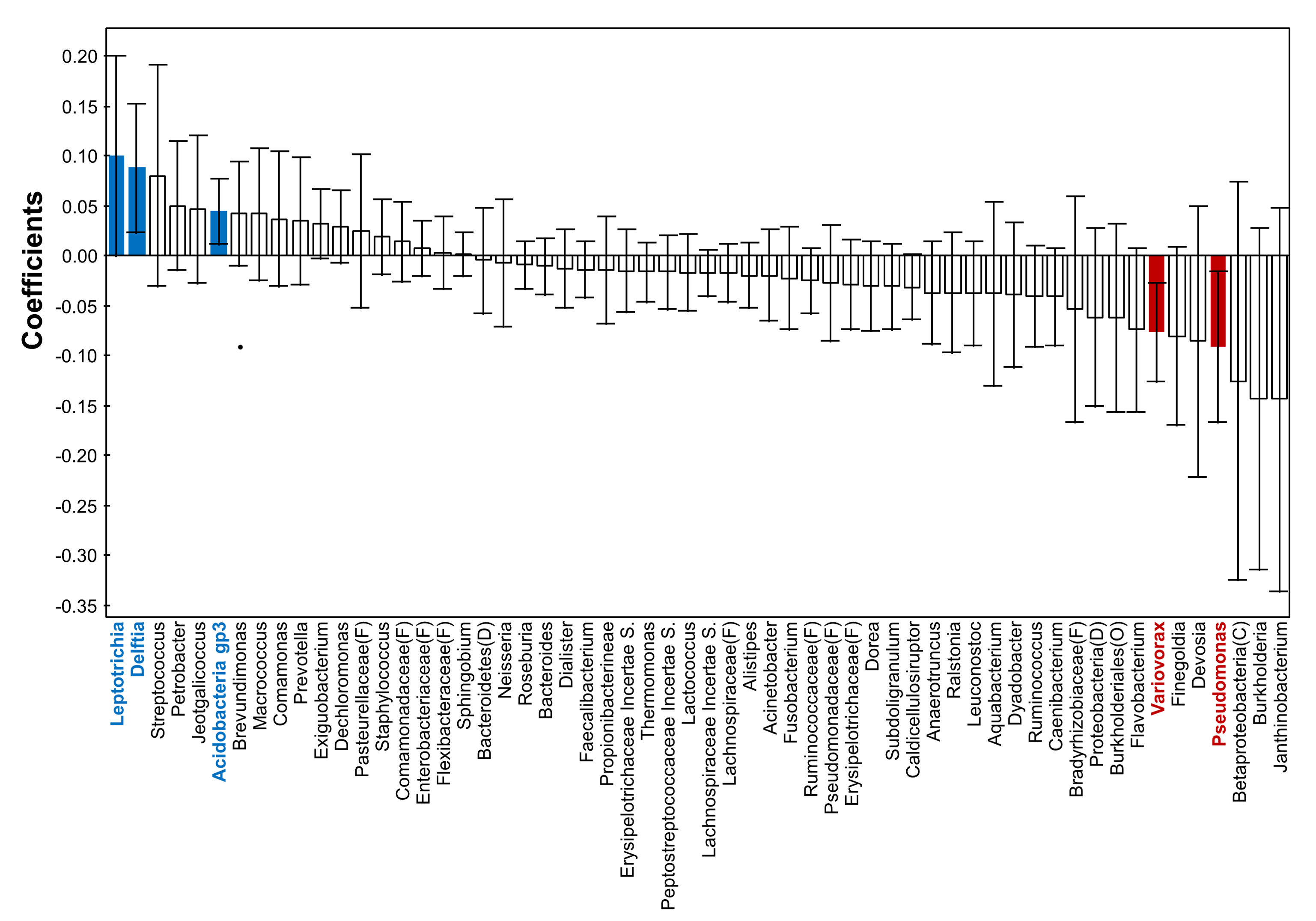

Supplement: Figure S3 — Coefficient plot of the bacterial profiles of poorly attractive (PA) and highly attractive (HA) individuals. Partial least squares-discriminant analysis (PLS-DA) coefficient plot based on the relative abundance of bacterial genera in the microbiota profiles of PA and HA individuals. Genera with significantly positive (>0) or negative (<0) PLS regression coefficients (i.e. no overlap between the 95% confidence interval indicated and the horizontal axis) contribute significantly to the prediction of the HA individuals (blue bars) or PA individuals (red bars), respectively. Coefficients were scaled and centred. Some sequences could only be identified to division (D), class (C), order (O) or family (F). (TIF) [file pone.0028991.s003.tif]
