# Supplementary material for: Composition of Human Skin Microbiota Affects Attractiveness to Malaria Mosquitoes
Source: PLoS One. 2011 Dec 28;6(12):e28991. doi: 10.1371/journal.pone.0028991 (PMC3247224; doi:10.1371/journal.pone.0028991)
Supplement: Table S1 — Ingredients of selective media used to determine the diversity of the human skin microbiota samples. (DOC) [file pone.0028991.s004.doc]

**Table S1. Ingredients of selective medium used to determine the diversity of the human skin microbiota samples.**

| **Selective medium** | **Ingredient** | **Amount per litre** |
| --- | --- | --- |
| Aerobic coryneform | Blood agar base no. 2 | 39.5 g |
|  | Yeast extract | 3 g |
|  | Glucose | 2 g |
|  | Tween 80 | 5 mL |
|  | Defibrinated horse blood | 50 mL |
|  | Phosphomycin | 500 mg |
|  |  |  |
| Micrococci | Nutrient agar | 28 g |
|  | Furazolidone | 6 mg |
|  |  |  |
| Propionibacteria | Fastidious anaerobe agar | 46 g |
|  | Defibrinated horse blood | 50 mL |
|  | Furazolidone | 6 mg |
|  |  |  |
| Staphylococci | Tryptone | 10 g |
|  | Laboratory Lemco powder | 5 g |
|  | Yeast extract | 3 g |
|  | Agar no. 1 | 13 g |
|  | Sodium pyruvate | 10 g |
|  | Glycine | 0.5 g |
|  | Potassium thiocyanate | 22.5 g |
|  | Disodium hydrogen orthophosphate dehydrate | 1.2 g |
|  | Sodium dihydrogen phosphate | 0.67 g |
|  | Lithiumchloride | 2 g |
|  | Glycerol | 10 mL |
|  | Sodiumazide | 20 mg |
|  | Egg yolk emulsion pH 7.2 | 30 mL |

Ingredients and methods as described before [12]. Media and plates were custom made at Tritium, The Netherlands.
